# Risk factors for mortality and progression to severe COVID-19 disease in the Southeast region in the United States: A report from the SEUS Study Group

**DOI:** 10.1017/ice.2020.1435

**Published:** 2021-01-11

**Authors:** Athena L. V. Hobbs, Nicholas Turner, Imad Omer, Morgan K. Walker, Ronald M. Beaulieu, Muhammad Sheikh, S. Shaefer Spires, Christina T. Fiske, Ryan Dare, Salil Goorha, Priyenka Thapa, John Gnann, Jeffrey Wright, George E. Nelson

**Affiliations:** 1 Department of Pharmacy, Methodist University Hospital, Memphis, Tennessee; 2 Department of Medicine, Duke University Medical Center, Durham, North Carolina; 3 Department of Medicine, Baptist Memorial Hospital-Memphis, Tennessee; 4 Department of Medicine, Louisiana State University Health Sciences Center, New Orleans, Louisiana; 5 Department of Medicine, Vanderbilt University Medical Center, Nashville, Tennessee; 6 Department of Medicine, University of Arkansas for Medical Sciences Medical Center, Little Rock, Arkansas; 7 Department of Medicine, The Medical University of South Carolina University Medical Center, Charleston, South Carolina

## Abstract

**Objective:**

Identify risk factors that could increase progression to severe disease and mortality in hospitalized SARS-CoV-2 patients in the Southeast region of the United States.

**Design, setting, and participants:**

Multicenter, retrospective cohort including 502 adults hospitalized with laboratory-confirmed COVID-19 between March 1, 2020, and May 8, 2020 within 1 of 15 participating hospitals in 5 health systems across 5 states in the Southeast United States.

**Methods:**

The study objectives were to identify risk factors that could increase progression to hospital mortality and severe disease (defined as a composite of intensive care unit admission or requirement of mechanical ventilation) in hospitalized SARS-CoV-2 patients in the Southeast United States.

**Results:**

In total, 502 patients were included, and 476 of 502 (95%) had clinically evaluable outcomes. The hospital mortality rate was 16% (76 of 476); 35% (177 of 502) required ICU admission and 18% (91 of 502) required mechanical ventilation. By both univariate and adjusted multivariate analyses, hospital mortality was independently associated with age (adjusted odds ratio [aOR], 2.03 for each decade increase; 95% confidence interval [CI], 1.56-–2.69), male sex (aOR, 2.44; 95% CI, 1.34–4.59), and cardiovascular disease (aOR, 2.16; 95% CI, 1.15–4.09). As with mortality, risk of severe disease was independently associated with age (aOR, 1.17 for each decade increase; 95% CI, 1.00–1.37), male sex (aOR, 2.34; 95% CI, 1.54–3.60), and cardiovascular disease (aOR, 1.77; 95% CI, 1.09–2.85).

**Conclusions:**

In an adjusted multivariate analysis, advanced age, male sex, and cardiovascular disease increased risk of severe disease and mortality in patients with COVID-19 in the Southeast United States. In-hospital mortality risk doubled with each subsequent decade of life.

Severe acute respiratory syndrome coronavirus 2 (SARS-CoV-2) and the disease it causes, novel coronavirus disease 2019 (COVID-19), was first identified in Wuhan, China, in December 2019, but it quickly spread and was declared a global pandemic by the World Health Organization (WHO) on March 11, 2020.^1,2^ Several early studies demonstrated various pre-existing conditions to be associated with severe disease^3,4^ and mortality,^5,6^ and in the United States, data have shown that between 14% and 30% of hospitalized patients require ICU level care and that older age and male sex associated with increased risk for ICU admission and mortality.^7,8^

The Southeast region of the United States reports the highest rates of obesity, diabetes, hypertension, and smoking in the nation,^9-12^ yet data about the effects of COVID-19 in this region are limited to 2 studies. Both relied on administrative data from a single health system; one study did not evaluate mortality as an outcome and the other reported outcomes that were not unadjusted for underlying comorbidities.^13,14^ A wide, continually expanding range of clinical features have been reported, and continued description of emerging clinical characteristics and outcomes in diverse, underrepresented populations are needed.^7,8,15^

The purpose of this multicenter, retrospective cohort is to determine risk factors associated with severe clinical outcomes from COVID-19 in a patient population with a high prevalence of comorbidities; determining these factors can help clinicians identify and triage high-risk patients at risk for adverse outcomes associated with COVID-19 in the Southeast United States.

## Methods

This multicenter, retrospective cohort study was conducted through the Southeast United States (SEUS) study group, which encompasses 15 hospitals, including both academic and community healthcare settings, ranging from 25 to 1,046 licensed beds within 5 healthcare systems across 5 states: Arkansas, Louisiana, Mississippi, North Carolina, and Tennessee. Adult hospitalized patients with a positive SARS-CoV-2 real-time reverse transcriptase-polymerase chain reaction (RT-PCR) assay using nasopharyngeal swab specimens collected during or immediately prior to hospital admission were identified through a review of positive laboratory values and were enrolled consecutively between March 1, 2020, through May 8, 2020. Demographic data, exposure risk, underlying conditions, clinical presentation, medications, nonmedication therapy (eg, mechanical ventilation), and outcome data were collected for eligible patients through electronic medical record (EMR) review and were entered into a REDCap database.^16^

The primary outcome was in-hospital mortality in patients with COVID-19 in the Southeast United States. Secondary outcomes included severe disease (defined as a composite of ICU admission or requirement of mechanical ventilation) and other clinical characteristics and epidemiological features associated with COVID-19 in the Southeast United States.

### Underlying conditions

Chronic kidney disease was defined as Kidney Disease Outcomes Quality Initiative (KDOQI) stages 3 through 5 for estimated glomerular filtration rate (eGFR) < 60 mL/min. Peripheral vascular disease included venous stenosis, deep vein thrombosis, and chronic venous stasis. Connective tissue disease included osteoarthritis and rheumatoid arthritis. Liver disease was defined as chronic hepatitis, portal hypertension, or cirrhosis. Although they were evaluated separately, we also analyzed a composite of patients with immune compromising conditions, cardiovascular disease, and pulmonary disease to reduce the impact of a small sample size. Immune compromise included active cancer or hematologic malignancy, history of solid-organ transplantation, HIV/AIDS, or autoimmune diseases with active use of immunosuppressive medications. Cardiovascular disease included congestive heart failure, coronary artery disease, arrhythmia, or other cardiovascular disease. Pulmonary disease was defined as asthma, COPD, or other chronic lung disease.

### Statistical analysis

Descriptive summary data were reported as counts and percentages for categorical variables, and mean (± standard deviation [SD] for normally distributed) or median (including interquartile range [IQR] for nonnormally distributed) for continuous variables. Outcomes analyses were limited to patients who were either discharged or deceased by the end of the inclusion period (May 8, 2020). Patients were analyzed across age groups defined in 10-year increments (eg, 20–29 years) through 99 years, accounting for all patients. Comparisons of categorical data between groups were analyzed using the χ^2^ test or the Fisher exact test when appropriate, and continuous data were analyzed using the Student *t* test or the Mann-Whitney U test when appropriate. Analysis of variance (ANOVA) was used for comparisons of continuous data across multiple groups when applicable.

We developed 3 separate multivariable logistic regression models to evaluate risk factors for hospital mortality or severe disease and to analyze any association between hospital mortality and treatment with azithromycin or hydroxychloroquine. Full models included all of the following a priori selected clinical variables: age, race, ethnicity, sex, body mass index (BMI), cardiovascular disease, pulmonary disease, liver disease, kidney disease, history of stroke, hypertension, diabetes, active smoking, peripheral vascular disease, connective tissue disease, or immune compromise. For each model, covariates were assessed for collinearity by assuring variance inflation factors <2, and continuous variables were assessed for nonlinearity by comparing to a restricted cubic spline model. Potential institution-specific differences in mortality were assessed, and no significant independent difference was detected when included as a covariate. For the treatment effect model, subjects participating in any clinical studies were excluded both to protect study integrity and because receipt of active study drug versus placebo could not be verified. All statistical analyses were conducted with R version 3.6.0 software (R Foundation for Statistical Computing, Vienna, Austria). All statistical tests were 2-sided with an α level of 0.05. All confidence intervals (CIs) were 95%.

## Results

In total, 502 adult hospitalized patients were included in the analysis. All COVID-19 cases were index cases and were confirmed by RT-PCR on specimens collected from the nasopharynx.^17^ Median age was 62 years (IQR, 49–71). Of these 502 patients, 225 (45%) were female; 287 (57%) were African American (black), 190 (38%) Caucasian (white), and 21 (4.2%) reported Hispanic ethnicity (Table 1). Patients were treated in facilities across 5 states in the Southeast: 163 (32%) in Tennessee, 127 (25%) in Mississippi, 80 (16%) in Louisiana, 76 (15%) in North Carolina, and 56 (11%) in Arkansas (Table 2). Also, 50% of patients received hydroxychloroquine at some point during their hospitalization, primarily reflecting the practice of a single health system at the time (Table 1).

**Table 1.** Baseline Characteristics of Hospitalized Patients with COVID-19 by Disease Severity and Mortality, Southeast United States

Characteristic	All Patients (n = 502)	Disease Severity			Mortality
		Nonsevere (n = 323)	Severe^a^ (n = 179)	Survived (n = 400)	Died (n = 76)
Age, median y (IQR)	62 (49–71)	58 (46–70)	65 (57–74)	58 (47–69)	72 (65–79)
Sex, female, no./total (%)	225 (44.8)	165/323 (51.1)	60/179 (33.5)	188/400 (47.0)	25/76 (32.9)
**Race, no./total (%)**					
Asian	6 (1.2)	4/323 (1.2)	2/179 (1.1)	5/400 (1.2)	0/76 (0)
African American	287 (57.2)	186/323 (57.6)	101/179 (56.4)	235/400 (58.8)	34/76 (44.7)
Caucasian	190 (37.8)	120/323 (37.2)	70/179 (39.1)	143/400 (35.8)	42/76 (55.3)
Other	19 (3.8)	13/323 (4.0)	6/179 (3.4)	17/400 (4.2)	0/76 (0)
**Ethnicity, no./total (%)**					
Hispanic/Latino	21 (4.2)	14/323 (4.3)	7/179 (3.6)	19/400 (4.8)	0/76 (0)
Non-Hispanic/Latino	471 (93.8)	303/323 (93.8)	168/179 (93.9)	372/400 (93.0)	76/76 (100.0)
Unknown	10 (2.0)	6/323 (1.9)	4/179 (2.2)	9/400 (2.2)	0/76 (0)
Weight, median kg (IQR)	87.8 (74.8–106.0)	86.2 (70.7–101.7)	89.8 (74.3–105.3)	88.5 (72.9–104.2)	85.7 (68.9–102.4)
Body mass index, median (IQR)	30.5 (25.8–35.7)	30.7 (25.9–35.5)	29.7 (24.7–34.8)	30.7 (25.9–35.5)	28.3 (22.2–34.4)
**Body mass index categories**					
Underweight (<18.5)	12/498 (2.4)	9/322 (2.8)	3/176 (1.7)	9/397 (2.3)	3/75 (4.0)
Normal (18.5 to <25)	98/498 (19.7)	65/322 (20.3)	33/176 (18.5)	75/397 (18.9)	18/75 (24.0)
Overweight (25 to <30)	131/498 (26.3)	77/322 (24.1)	54/176 (30.3)	99/397 (24.9)	22/75 (29.3)
Obese (30 to <40)	188/498 (37.8)	126/322 (39.4)	62/176 (34.8)	160/397 (40.3)	21/75 (28.0)
Morbidly obese (>40)	69/498 (13.9)	43/322 (13.4)	26/176 (14.6)	54/397 (13.6)	11/75 (14.7)
**Comorbidities, no./total (%)**					
Any cardiovascular disease	158/502 (31.5)	83/323 (25.7)	75/179 (41.9)	102/400 (25.5)	48/76 (63.2)
Congestive heart failure	71/502 (14.1)	37/323 (11.5)	34/179 (19.0)	43/400 (10.8)	23/76 (30.3)
Coronary vascular disease	75/502 (14.9)	39/323 (12.1)	36/179 (20.1)	51/400 (12.8)	21/76 (27.6)
Cardiac arrhythmia	55/502 (11.0)	24/323 (7.4)	31/179 (17.3)	31/400 (7.8)	22/76 (28.9)
Other cardiovascular disease	38/502 (7.6)	23/323 (7.1)	15/179 (8.4)	26/400 (6.5)	8/76 (10.5)
Peripheral vascular disease	38/502 (7.6)	21/323 (6.5)	17/179 (9.5)	28/400 (7.0)	10/76 (13.2)
Cerebrovascular disease	57/502 (11.4)	35/323 (10.8)	22/179 (12.3)	41/400 (10.2)	13/76 (17.1)
Hypertension	313/502 (62.4)	193/323 (59.8)	120/179 (67.0)	242/400 (60.5)	56/76 (73.7)
Diabetes mellitus	169/502 (33.7)	98/323 (30.3)	71/179 (39.7)	125/400 (31.2)	30/76 (39.5)
Any pulmonary disease	93/502 (18.5)	61/323 (18.9)	32/179 (17.9)	72/400 (18.0)	19/76 (25.0)
Asthma	49/502 (9.8)	35/323 (10.8)	14/179 (7.8)	43/400 (10.8)	5/76 (6.6)
COPD	33/502 (6.6)	19/323 (5.9)	14/179 (7.8)	21/400 (5.2)	12/76 (15.8)
Other pulmonary disease	21/502 (4.2)	14/323 (4.0)	8/179 (4.5)	14/400 (3.5)	6/76 (7.9)
Smoking	22/502 (4.4)	12/323 (3.7)	10/179 (5.6)	18/400 (4.5)	3/76 (3.9)
Chronic kidney disease	56/502 (11.2)	32/323 (9.9)	24/179 (13.4)	37/400 (9.2)	16/76 (21.1)
End-stage renal disease	30/502 (6.0)	17/323 (5.3)	13/179 (7.3)	23/400 (5.8)	3/76 (3.9)
Liver disease	13/502 (2.6)	5/323 (1.5)	8/179 (4.5)	9/400 (2.2)	3/76 (3.9)
Connective tissue disease	32/502 (6.4)	16/323 (5.0)	16/179 (8.9)	17/400 (4.2)	12/76 (15.8)
Immune compromised	62/502 (12.4)	34/323 (10.5)	28/179 (15.6)	46/400 (11.5)	10/76 (13.2)
Cancer (solid tumor)	26/502 (5.2)	11/323 (3.4)	15/179 (8.4)	16/400 (4.0)	7/76 (9.2)
Hematologic malignancy	10/502 (2.0)	9/323 (2.8)	1/179 (0.6)	8/400 (2.0)	0/76 (0)
Solid organ transplant	9/502 (1.8)	4/323 (1.2)	5/179 (2.8)	7/400 (1.8)	1/76 (1.3)
AIDS	0/502 (0)	0/323 (0)	0/179 (0)	0/400 (0)	0/76 (0)
HIV	7/502 (1.4)	6/323 (1.9)	1/179 (0.6)	7/400 (1.8)	0/76 (0)
Immunosuppressive meds	14/502 (2.8)	7/323 (2.2)	7/179 (3.9)	12/400 (3.0)	2/76 (2.6)
**Treatment, no./total. (%)**					
Azithromycin	246/496 (49.6)	157/319 (49.2)	89/177 (50.3)	203/394 (51.5)	33/76 (43.4)
Convalescent plasma	6/496 (1.2)	0/319 (0)	6/177 (3.4)	1/394 (0.3)	2/76 (2.6)
Hydroxychloroquine	245/490 (50.0)	154/317 (48.6)	91/173 (52.6)	200/390 (51.3)	36/75 (48.0)
Lopinavir	0/496 (0)	0/320 (0)	0/176 (0)	0/394 (0)	0/76 (0)
Remdesivir	6/492 (1.2)	0/316 (0)	6/176 (3.4)	3/392 (0.8)	0/76 (0)
Tocilizumab	23/497 (4.6)	3/320 (0.9)	20/177 (11.3)	12/395 (3.0)	8/76 (10.5)
Study participant	23/446 (5.2)	11/285 (3.9)	12/161 (7.5)	16/354 (4.5)	5/66 (7.6)
Length of stay, median d (IQR)	5.9 (3.0–10.1)	4.7 (2.5–6.9)	10.0 (4.8–15.2)	5.9 (2.6–9.2)	7.3 (2.8–11.8)

Note. IQR, interquartile range; COPD, chronic obstructive pulmonary disease; HIV, human immunodeficiency virus; AIDS, acquired immunodeficiency syndrome.

a Severe disease, defined as a composite of ICU admission or requirement of mechanical ventilation.

**Table 2.** Presentation Characteristics of Hospitalized Patients with COVID-19 by Disease Severity and Mortality, Southeast United States

Patient Presentation	No./Total (N = 502) (%)
**Epidemiologic risk factors for COVID-19**	
Admitted from congregate setting^a^	155/502 (31)
Known exposure to COVID-positive individual	199/502 (40)
Healthcare worker	35/502 (7)
Physician	6/35 (17)
Nurse	11/35 (31)
Other	18/35 (51)
**Evaluation location**	
Patients evaluated prior to admission	218/502 (43)
ED evaluation^b^	133/502 (61)
COVID assessment site	35/502 (16)
Other	50/502 (23)
**Testing timing**	
Patients with positive SARS-CoV-2 assay during admission	366/502 (73)
Mean time to positive test after admission, days	1.6±2.9
Patients with positive SARS-CoV-2 assay prior to admission	79/502 (16)
Mean time to positive test prior to admission, d	0.7±2.3
Not reported	57/502 (11)
**Hospitalized location, state**	
Arkansas	56/502 (11)
North Carolina	76/502 (15)
Louisiana	80/502 (16)
Mississippi	127/502 (25)
Tennessee	163/502 (32)
**Clinical presentation**	
Fever (Tmax >38°C in house or prior to admission)	352/502 (70)
Productive cough	80/502 (16)
Dry cough	277/502 (55)
Dyspnea	317/502 (63)
Night sweats	27/502 (5)
Chills	94/502 (19)
Sore throat	36/502 (7)
Fatigue	187/502 (37)
Anorexia	80/502 (16)
Myalgia	142/502 (28)
Nasal congestion	45/502 (9)
Rhinorrhea	37/502 (7)
Nausea	105/502 (21)
Vomiting	66/502 (13)
Diarrhea (>3 episodes of loose stool within previous 24 h)	104/502 (21)
Abdominal pain	49/502 (10)
Headache	70/502 (14)
Rash	2/502 (0)
Anosmia (loss of smell)	25/502 (5)
Ageusia (loss of taste) or dysgeusia (altered taste)	36/502 (7)
Psychataxia (difficulty concentrating)	29/502 (6)
Pleurisy/chest pain	57/502 (11)
No. of patients reporting symptoms prior to admission	471/502 (94)
Mean duration of symptoms prior to admission, d	5.2±4.5

Note. ED, emergency department.

a Congregate setting is a school, workplace, prison, etc.

Baseline risk factors for COVID-19 included known exposure to a COVID-19–positive individual (199 of 502, 40%) and presentation from a congregate setting (eg, nursing facility or prison (155 of 502, 31%). Of 502 a COVID-19–positive individuals, only 35 (7%) were healthcare workers; 11 of these (31%) were nurses; 6 (17%) were physicians; and 18 (51%) identified themselves as healthcare workers without designated role. Of these 502 patients 218 (43%) were evaluated prior to admission, and 133 (61%) of these 218 were assessed in an emergency department (ED), though 366 of the total 502 patients (73%) had a positive SARS-CoV-2 RT-PCR during admission. The mean time between a positive assay and admission was 0.7 days (± 2.3) for those with a positive test prior to admission. Also, 471 (94%) were symptomatic upon admission. Fever was the most commonly reported symptom (352, 70%), followed by dyspnea (317, 63%), dry cough (277, 55%), fatigue (187, 37%), myalgia (142, 28%), nausea (105, 21%), and diarrhea defined as >3 episodes of loose stool within 24 hours (104, 21%). The mean duration of symptom onset prior to admission was 5.2 ± 4.5 days (Table 2).

Almost all patients (476 of 502, 95%) were either discharged or deceased by the end of the inclusion period and were included in outcome analyses. The overall hospital mortality rate was 16% (76 of 476). Moreover, 177 (35%) required intensive care unit (ICU) admission, and 91 (18%) required mechanical ventilation. Hospital length of stay was significantly longer for patients with severe disease (median, 10.0 days; IQR, 4.8–15.2) relative to nonsevere disease (median, 4.7 days; IQR, 2.5–6.9; *P* < .01).

Most patients (422 of 502, 84%) had at least 1 comorbidity, and 190 (38%) had at least 3 comorbidities. Hypertension was the most common comorbidity, affecting 313 patients (62%) More than half (257 of 498, 52%) were obese, with a median admission weight of 87.8 kg (IQR, 74.8–106.0) and median BMI of 30.5 (IQR, 25.8–35.7). One-third of patients had been diagnosed with diabetes mellitus (169 of 502, 34%), followed by coronary vascular disease and congestive heart failure, which affected 75 patients (15%) and 71 patients (14%), respectively. Notably, only 49 (10%) of patients had asthma, 33 (7%) had COPD, 22 (4%) were active smokers, and 21 (4%) had another chronic pulmonary disease (Table 1).

**Fig. 1.**
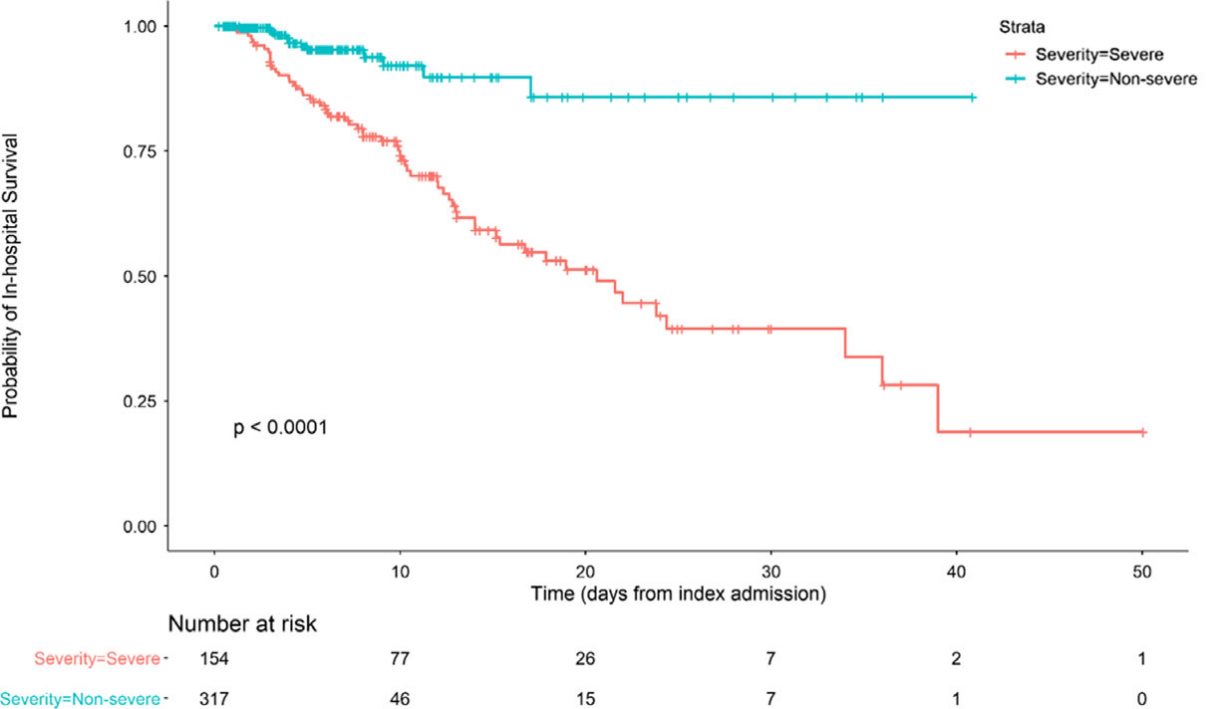
Kaplan-Meier curve stratified by disease severity. Severe disease is defined as a composite of ICU admission or requirement of mechanical ventilation.

Hospital mortality was independently associated with age (adjusted odds ratio [aOR], 2.03 for each decade increase in age; 95% confidence interval [CI], 1.56–2.69), male sex (aOR, 2.44; 95% CI, 1.34–4.59), cardiovascular disease (aOR, 2.16; 95% CI, 1.15–4.09), and connective tissue disease (aOR, 3.13; 95% CI, 1.25–7.81) by both univariate and adjusted analysis. Each comorbidity was individually assessed in univariate analysis; however, any statistical significance was lost after adjusting for additional comorbidities (Table 3 and Supplementary Tables S1 and S2 online). Patients at the extremes of both age and BMI tended to have higher mortality rates. For example, individuals aged 21–40 years and 61–80 years had higher mortality rates if they also had a BMI > 40 (1 of 13, 7.7% and 9 of 23, 39.1% respectively; Table 4). Similarly, mortality rates among individuals over the age of 80 appeared to be higher for underweight individuals versus those with a BMI between 18.5 and <25 (3 of 5, 60.0% vs 8 of 21, 38.1%). Small numbers of individuals were included in the most extreme combinations of age and BMI, which could have prevented comparisons from reaching statistical significance (age × BMI interaction test, *P* = .10) (Table 4). Neither azithromycin (aOR, 0.69; 95% CI, 0.35–1.37) nor hydroxychloroquine (aOR, 1.52; 95% CI, 0.77–3.06) were associated with any significant reduction in mortality by univariate or adjusted analysis (Table 1). Analysis was limited to these therapeutic agents due to limited availability of alternatives during the study period. Risk of severe disease, as with mortality, was independently associated with age (aOR, 1.17 for each decade increase in age; 95% CI, 1.00–1.37), male sex (aOR, 2.34; 95% CI, 1.54–3.60), and cardiovascular disease (aOR, 1.77; 95% CI 1.09–2.85) (Table 5). We also included a Kaplan-Meier mortality curve stratified by disease severity as a time to event analysis (Fig. 1).

**Table 3.** Risk of Mortality, Unadjusted and Adjusted Analyses of Hospitalized Patients with COVID-19, Southeast United States

Covariate	Univariate (Unadjusted)		Multivariate (Adjusted)	
	OR^a^ (95% CI^b^)	*P* Value	OR^a^ (95% CI^b^)	*P* Value
Age, per decade	2.13 (1.74–2.65)	<.01	2.03 (1.56–2.69)	<.01
Sex, male	1.81 (1.09–3.07)	.02	2.44 (1.34–4.59)	<.01
**Race/Ethnicity**				
White	Ref^c^	…	Ref^c^	…
Black	0.49 (0.30–0.81)	<.01	0.75 (0.41–1.36)	.34
**BMI** ^d^				
<18.5	1.39 (0.29–5.22)	.65	1.34 (0.22–6.54)	.73
18.5 to <25	Ref^c^	…	Ref^c^	…
25 to <30	0.93 (0.46–1.86)	.83	1.15 (0.51–2.64)	.73
30 to <40	0.55 (0.28–1.10)	.09	1.08 (0.48–2.50)	.85
≥40	0.85 (0.36–1.92)	.70	1.71 (0.58–4.87)	.33
**Comorbidities**				
Cardiovascular disease	5.01 (3.01–8.49)	<.01	2.16 (1.15–4.09)	.02
Pulmonary disease	1.52 (0.83–2.67)	.16	1.57 (0.78–3.12)	.20
Kidney disease	2.01 (1.09–3.57)	.02	0.89 (0.41–1.87)	.75
Stroke	1.81 (0.89–3.48)	.09	1.25 (0.55–2.72)	.58
Hypertension	1.83 (1.07–3.23)	.03	0.84 (0.42–1.69)	.61
Diabetes mellitus	1.43 (0.86–2.37)	.16	1.43 (0.75–2.74)	.28
Peripheral vascular	2.01 (0.89–4.22)	.07	1.25 (0.49–3.06)	.63
Connective tissue disease	4.22 (1.89–9.21)	<.01	3.13 (1.25–7.81)	.01
Liver disease	1.79 (0.39–6.15)	.39	2.10 (0.39–8.96)	.34
Smoking	0.87 (0.20–2.66)	.83	1.11 (0.22–4.14)	.88
Immune compromised	1.17 (0.53–2.34)	.68	1.58 (0.63–3.72)	.31

Note. OR, odds ratio; CI, confidence interval; Ref, reference; BMI, body mass index.

**Table 4.** Matrix of Mortality Rate by Combined Age/BMI Categories deaths/total (%) of Hospitalized Patients with COVID-19, Southeast United States

Age Group, y	BMI Category, No./Total (%)				
	<18.5	18.5 to <25	25 to <30	30 to <40	≥40
<21	0/0 (0)	0/1 (0)	0/0 (0)	0/1 (0)	0 (0)
21–40	0/0 (0)	0/10 (0)	0/12 (0)	0/31 (0)	1/13 (7.7)
41–60	0/2 (0)	1/24 (4.2)	2/44 (4.5)	3/72 (4.2)	1/29 (3.4)
61–80	0/5 (0)	9/42 (21.4)	13/60 (21.7)	16/73 (21.9)	9/23 (39.1)
>80	3/5 (60.0)	8/21 (38.1)	7/15 (46.7)	2/11 (18.2)	0/4 (0)

Note. BMI, body mass index.

**Table 5.** Risk of Severe Disease,^a^ Unadjusted and Adjusted Analyses of Hospitalized Patients With COVID-19, Southeast United States

Covariate	Univariate (Unadjusted)		Multivariate (Adjusted)	
	OR (95% CI)	*P* Value	OR (95% CI)	*P* Value
Age, per decade	1.34 (1.20–1.52)	<.01	1.17 (1.00–1.37)	.05
Sex, male	2.02 (1.39–2.94)	<.01	2.34 (1.54–3.60)	<.01
**Race/Ethnicity**				
White	Ref^c^	…	Ref^c^	…
Black	0.81 (0.56–1.19)	.28	1.14 (0.75–1.76)	.54
**BMI**				
	0.66 (0.14–2.37)	.54	0.84 (0.17–3.31)	.81
18.5 to <25	Ref^c^	…	Ref^c^	…
25 to <30	1.38 (0.80–2.39)	.24	1.64 (0.90–3.04)	.11
30 to <40	0.97 (0.58–1.64)	.91	1.35 (0.75–2.45)	.32
≥40	1.19 (0.62–2.26)	.59	1.94 (0.93–4.12)	.08
**Comorbidities**				
Cardiovascular disease	2.52 (1.71–3.73)	<.01	1.77 (1.09–2.85)	.02
Pulmonary disease	0.90 (0.56–1.44)	.67	0.87 (0.51–1.48)	.62
Kidney disease	1.40 (0.86–2.26)	.17	0.86 (0.48–1.51)	.60
Stroke	1.37 (0.78–2.38)	.27	0.97 (0.51–1.81)	.93
Hypertension	1.43 (0.98–2.09)	.06	1.08 (0.67–1.74)	.75
Diabetes mellitus	1.35 (0.92–1.98)	.12	1.19 (0.75–1.87)	.46
Peripheral vascular	1.46 (0.74–2.83)	.27	1.07 (0.50–2.25)	.87
Connective tissue disease	2.12 (1.03–4.44)	.04	1.68 (0.76–3.71)	.20
Liver disease	2.58 (0.85–8.64)	.10	2.54 (0.79–8.93)	.12
Smoking	1.33 (0.55–3.13)	.52	1.53 (0.58–3.92)	.38
Immune compromised	1.58 (0.92–2.69)	.10	1.76 (0.97–3.18)	.06

Note. OR, odds ratio; CI, confidence interval; Ref, reference; BMI, body mass index.

a Severe disease, defined as a composite of ICU admission or requirement of mechanical ventilation.

## Discussion

This multicenter study is the first to report risk factors for severe disease and death in hospitalized patients with COVID19 in the Southeast region of the United States, which encompasses a patient population with high rates of underlying comorbidities. Patients included in these analyses represent a large geographic region underreported in current studies and hospitalized in both community and academic institutions from 5 separate health systems in 5 different states in the Southeast. This broad representation, in addition to extensive chart review, allowed for in-depth, patient-level detail with adjusted risk factor analyses not currently available in many larger-scale epidemiological studies using administrative data sets that often have high rates of missing data.

In a multivariate analysis, age emerged as an independent risk factor for in-hospital mortality and severe COVID-19 disease with mortality risk doubling with each consecutive decade of life. Age was also an independent risk factor for severe disease, increasing 1.17 fold (95% CI, 1.00–1.37; *P* = .05) for each subsequent decade. These findings are consistent with recent observational cohort studies.^6,18-20^ However, despite almost half of our cohort being ≤60 years, only 8 deaths occurred in this group, resulting in a lower mortality rate in this younger cohort compared to earlier reports.^7,20^ Furthermore, few patients (n = 68) were ≤40 years, which accounted for only 1 death. We identified age as an important prognostic factor for worse outcomes and compounded risk with increasing age in patients with COVID-19 using a robust risk assessment based on 3 different multivariate statistical models that accounted for a population with multiple comorbidities and diverse demographics.

Most inpatients with COVID-19 in our study were obese, with a median BMI of 30.5, consistent with previous studies demonstrating increased rates of obesity among those hospitalized with COVID-19.^7,21,22^ In our study, obesity was not an independent predictor of severe disease or mortality in a multivariate analysis after controlling for other comorbidities such as diabetes and cardiovascular disease. In contrast, previous studies found morbid obesity (BMI >40) to be associated with increased risk of COVID-19 critical illness.^23^

Racial disparities among patients diagnosed with COVID-19 in the United States have been widely publicized. One study found a positive correlation between the prevalence of racial minorities and COVID-19 case and mortality rates; however, these results were not adjusted for potential confounders.^24^ Multiple case series have shown that self-identified African American, black, or black non-Hispanic race is an independent risk factor for hospital admission.^13,14,25^ A large retrospective cohort in Louisiana found that black, non-Hispanic patients accounted for 70.4% of COVID-19 cases, 76.9% of hospital admissions, and 70.6% of deaths despite constituting only 31% of the total health system population.^14^ Consistent with our findings, those investigators found that race was not independently associated with mortality after adjusted analysis.

Most of our patients self-identified as either Caucasian (white) or African American (black). Only 5% identified as Asian or “other,” and Hispanic ethnicity was infrequently reported limiting robust analysis for these populations. Data were manually abstracted using chart review and did not rely on automatic abstraction, which provides confidence in these results. Our analysis included patients from the initial phase of the pandemic and does not account for the changing demographics of those hospitalized with COVID-19 in the Southeast over time. Personal communications at each of the sites note a larger proportion of Hispanic patients now hospitalized, with increased community spread and localized outbreaks identified in certain industries (eg, food and meat processing). Our study was restricted to the first 10 weeks of the pandemic in the Southeast, before widespread community prevalence. This factor may account for the low Hispanic population and older age of our study cohort, despite the multicenter design.

We found that blacks represented a large portion of COVID-19 hospital admissions (57.2%) despite comprising only 16.1%–37.6% of the population in the states represented in this study.^26^ Univariate analysis suggested increased in-hospital mortality among white patients despite no difference in rates of severe disease. However, this finding lost statistical significance after controlling for age and comorbid conditions. The CDC has suggested that the increased risk of hospitalization among racial minorities may be due to living, work, and health circumstances including a greater burden of concomitant comorbidities such as diabetes, hypertension, and obesity.^27^ Other potential hypotheses include differential employment in essential jobs, reliance on public transportation, and residence in Southern states where “shelter at home” orders were delayed or prematurely lifted.^28^

Our results do not demonstrate any significant correlation between smoking, asthma, or COPD and increased mortality or disease severity from COVID-19, though we recognize the small overall sample sizes for each being 4%, 10%, and 7%, respectively (Supplementary Tables S1 and S2 online). Previous studies have yielded conflicting results regarding the association between smoking and COVID-19 disease severity or death.^29,30^ Interestingly, some have hypothesized various explanations for these observations ranging from modulation of pathogenesis to differential practice of preventive measures.^31-33^ These explanations should be interpreted with caution given the inability to establish causality.

History of cardiovascular disease was a significant risk factor in COVID-19 similar to prior studies, more than doubling the risk for mortality and nearly doubling the risk for severe disease.^3,34^ Pre-existing cardiovascular disease has been associated with worse clinical outcomes in patients who develop COVID-19.^35^ Interestingly, although hypertension was the most common comorbidity reported and appeared to increase risk of mortality in univariate analysis, this finding did not persist in the multivariate analysis. Contradictory to some previous studies, neither our univariate nor our multivariate analysis showed that hypertension increased risk of severe COVID-19.^3,29^

This study has several limitations, mainly due to the retrospective design. They include the evolving nature of the characteristics of the SARS-CoV-2 outbreak during the period of consecutive patient data collection, limited availability of testing, and variability of treatment algorithms across sites. The timing of the outbreak was delayed in some of the Southeast states compared to other states, and each hospital population represents only the initial population of hospitalized COVID-19 patients likely before extensive community spread. This phenomenon may have skewed the study toward more vulnerable populations including those in long-term care facilities and other congregate settings where early spread was recognized. However, the multistate design including community-based and tertiary-care hospital representation provided broad representation across the Southeast region of the United States and limited bias toward a single group. We did not collect data on initial oxygen requirement that, in retrospect, could help identify patients who might require mechanical ventilation. Additionally, the reduced availability of COVID-19 testing at the beginning of the pandemic, and potential underreporting and misclassification of COVID-19 cases attributed to other circulating seasonal respiratory viruses, may have influenced complete case ascertainment. All sites used FDA-approved molecular diagnostic tests to perform RT-PCR; however, the different RNA gene targets used to identify SARS-CoV-2 can vary in their sensitivity.^36,37^ Furthermore, small sample size in some of the stratified analysis may have limited our ability to detect a difference between groups, such as individual underlying conditions (eg, COPD). The loss of effect upon multivariate adjustment likely suggests the results being underpowered and the presence of some confounding between comorbidities. The absence of a clear link between obesity and mortality in our study does not rule out such a possibility. Rather, because of the high prevalence of obesity overall and the low number of subjects and events occurring at more extreme BMIs, our study may simply have been underpowered to detect such an effect. There is also likely high variability in treatments administered in the beginning of this pandemic due to the lack of approved treatments available. However, this limitation is likely minimal because few treatment options with proven clinical benefit, such as remdesivir, were widely available at the time of data collection for this study.

This multicenter cohort provides a robust analysis adjusting for potential confounders to describe risk factors associated with hospital mortality and severe disease from COVID-19 in the Southeast region of the United States. Increasing and advanced age, male sex, and cardiovascular disease are all independent risk factors for mortality and progression to severe disease. Furthermore, mortality risk doubles with each successive decade of life. Notably, neither obesity nor race were significantly associated with either outcome after adjusted analyses. Understanding patients at risk offers vital information as the region braces for increased healthcare utilization, informs critical resource needs, and emphasizes awareness of patients at risk for severe clinical outcomes.

## Supplementary material

For supplementary material accompanying this paper visit http://dx.doi.org/10.1017/ice.2020.1435. click here to view supplementary material
