# Supplementary material for: Risk factors for mortality and progression to severe COVID-19 disease in the Southeast region in the United States: A report from the SEUS Study Group
Source: Infect Control Hosp Epidemiol. 2021 Jan 11:1–9. doi: 10.1017/ice.2020.1435 (PMC8576130; doi:10.1017/ice.2020.1435)

Supplementary Appendix

Table S1: Risk of Mortality, Unadjusted and Adjusted Analyses of Hospitalized Patients with COVID-19, Southeast United States; Individual Comorbidities

| **Covariate** | **Univariate (Unadjusted)** | | **Multivariate (Adjusted)** | |
| --- | --- | --- | --- | --- |
|  | OR^a^ (95% CI^b^) | p-value | OR^a^ (95% CI^b^) | p-value |
| **Age (per decade)** | 2.13 (1.74-2.65) | <0.01 | 2.07 (1.57-2.79) | <0.01 |
| **Gender (male)** | 1.81 (1.09-3.07) | 0.02 | 2.48 (1.34-4.75) | <0.01 |
| **Race/Ethnicity** |  |  |  |  |
| Caucasian | Ref^c^ | -- | Ref^c^ | -- |
| African American | 0.49 (0.30-0.81) | <0.01 | 0.74 (0.40-1.37) | 0.34 |
| **BMI**^d^ |  |  |  |  |
| <18.5 | 1.39 (0.29-5.22) | 0.65 | 1.45 (0.25-6.84) | 0.65 |
| 18.5 to <25 | Ref^c^ | -- | Ref^c^ | -- |
| 25 to <30 | 0.93 (0.46-1.86) | 0.83 | 1.26 (0.55-2.95) | 0.59 |
| 30 to <40 | 0.55 (0.28-1.10) | 0.09 | 0.99 (0.43-2.32) | 0.98 |
| ≥40 | 0.85 (0.36-1.92) | 0.70 | 1.67 (0.56-4.97) | 0.35 |
| **Co-morbidities** |  |  |  |  |
| Coronary vascular disease | 2.61 (1.44-4.64) | <0.01 | 0.89 (0.42-1.85) | 0.76 |
| Congestive heart failure | 3.60 (1.99-6.42) | <0.01 | 1.31 (0.59-2.86) | 0.50 |
| Cardiac arrhythmia | 4.85 (2.60-8.97) | <0.01 | 1.67 (0.76-3.63) | 0.19 |
| Other cardiovascular disease | 1.69 (0.69-3.74) | 0.22 | 1.20 (0.41-3.26) | 0.73 |
| COPD^e^ | 3.38 (1.55-7.12) | <0.01 | 1.58 (0.60-3.99) | 0.34 |
| Asthma | 0.58 (0.20-1.40) | 0.27 | 0.87 (0.25-2.56) | 0.81 |
| Other lung disease | 2.36 (0.81-6.11) | 0.09 | 3.35 (0.97-10.67) | 0.05 |
| Chronic kidney disease | 2.61 (1.34-4.93) | <0.01 | 1.44 (0.60-3.35) | 0.40 |
| End stage renal disease | 0.67 (0.16-2.00) | 0.53 | 0.32 (0.04-1.36) | 0.17 |
| Stroke | 1.81 (0.89-3.48) | 0.09 | 1.31 (0.57-2.89) | 0.52 |
| Hypertension | 1.83 (1.07-3.23) | 0.03 | 0.80 (0.39-1.63) | 0.53 |
| Diabetes mellitus | 1.43 (0.86-2.37) | 0.16 | 1.45 (0.75-2.83) | 0.27 |
| Peripheral vascular disease | 2.01 (0.89-4.22) | 0.07 | 1.40 (0.54-3.48) | 0.48 |
| Connective tissue disorder | 4.22 (1.89-9.21) | <0.01 | 2.88 (1.12-7.33) | 0.03 |
| Liver disease | 1.79 (0.39-6.15) | 0.39 | 2.14 (0.40-9.04) | 0.33 |
| Smoking | 0.87 (0.20-2.66) | 0.83 | 1.07 (0.21-4.19) | 0.93 |
| Immunocompromised | 1.36 (0.62-2.76) | 0.41 | 1.74 (0.68-4.20) | 0.23 |

^a^OR, odds ratio

^b^CI, confidence interval

^c^Ref, reference

^d^BMI, body mass index

^e^COPD, chronic obstructive pulmonary disease

Table S2: Risk of Severe Disease^+^, Unadjusted and Adjusted Analyses of Hospitalized Patients with COVID-19, Southeast United States; Individual Comorbidities

| **Covariate** | **Univariate (Unadjusted)** | | **Multivariate (Adjusted)** | |
| --- | --- | --- | --- | --- |
|  | OR^a^ (95% CI^b^) | p-value | OR^a^ (95% CI^b^) | p-value |
| **Age (per decade)** | 1.34 (1.20-1.52) | <0.01 | 1.14 (0.99-1.31) | 0.07 |
| **Gender (male)** | 2.02 (1.39-2.94) | <0.01 | 2.33 (1.53-3.60) | <0.01 |
| **Race/Ethnicity** |  |  |  |  |
| Caucasian | Ref^c^ | -- | Ref^c^ | -- |
| African American | 0.81 (0.56-1.19) | 0.28 | 1.15 (0.74-1.78) | 0.54 |
| **BMI**^d^ |  |  |  |  |
| <18.5 | 0.66 (0.14-2.37) | 0.54 | 0.86 (0.17-3.43) | 0.84 |
| 18.5 to <25 | Ref^c^ | -- | Ref^c^ | -- |
| 25 to <30 | 1.38 (0.80-2.39) | 0.24 | 1.82 (0.98-3.41) | 0.06 |
| 30 to <40 | 0.97 (0.58-1.64) | 0.91 | 1.37 (0.76-2.52) | 0.30 |
| ≥40 | 1.19 (0.62-2.26) | 0.59 | 2.00 (0.94-4.30) | 0.07 |
| **Co-morbidities** |  |  |  |  |
| Coronary vascular disease | 1.83 (1.11-3.01) | 0.02 | 1.08 (0.58-1.98) | 0.81 |
| Congestive heart failure | 1.81 (1.09-3.01) | 0.02 | 1.23 (0.64-2.33) | 0.53 |
| Cardiac arrhythmia | 2.61 (1.48-4.64) | <0.01 | 2.01 (1.04-3.92) | 0.04 |
| Other cardiovascular disease | 1.19 (0.59-2.33) | 0.61 | 0.98 (0.46-2.03) | 0.95 |
| COPD^e^ | 1.36 (0.65-2.76) | 0.40 | 0.95 (0.41-2.16) | 0.90 |
| Asthma | 0.70 (0.35-1.31) | 0.28 | 0.78 (0.36-1.59) | 0.50 |
| Other pulmonary disease | 1.12 (0.43-2.70) | 0.81 | 1.06 (0.38-2.81) | 0.91 |
| Chronic kidney disease | 1.41 (0.79-2.47) | 0.23 | 0.93 (0.47-1.81) | 0.83 |
| End stage renal disease | 1.41 (0.66-2.96) | 0.37 | 1.13 (0.47-2.65) | 0.78 |
| Stroke | 1.37 (0.78-2.38) | 0.27 | 0.92 (0.48-1.75) | 0.81 |
| Hypertension | 1.43 (0.98-2.09) | 0.06 | 1.11 (0.69-1.80) | 0.66 |
| Diabetes mellitus | 1.35 (0.92-1.98) | 0.12 | 1.17 (0.74-1.85) | 0.50 |
| Peripheral vascular disease | 1.46 (0.74-2.83) | 0.27 | 1.11 (0.51-2.36) | 0.79 |
| Connective tissue disorder | 2.12 (1.03-4.44) | 0.04 | 1.60 (0.72-3.58) | 0.25 |
| Liver disease | 2.58 (0.85-8.64) | 0.10 | 2.34 (0.71-8.36) | 0.17 |
| Smoking | 1.33 (0.55-3.13) | 0.52 | 1.54 (0.59-3.97) | 0.37 |
| Immunocompromised | 1.66 (0.94-2.94) | 0.08 | 1.93 (1.03-3.61) | 0.04 |

^+^Severe disease, defined as a composite of ICU admission or requirement of mechanical ventilation

^a^OR, odds ratio

^b^CI, confidence interval

^c^Ref, reference

^d^BMI body mass index

^e^COPD, chronic obstructive pulmonary disease

Table S3 Mortality by Age


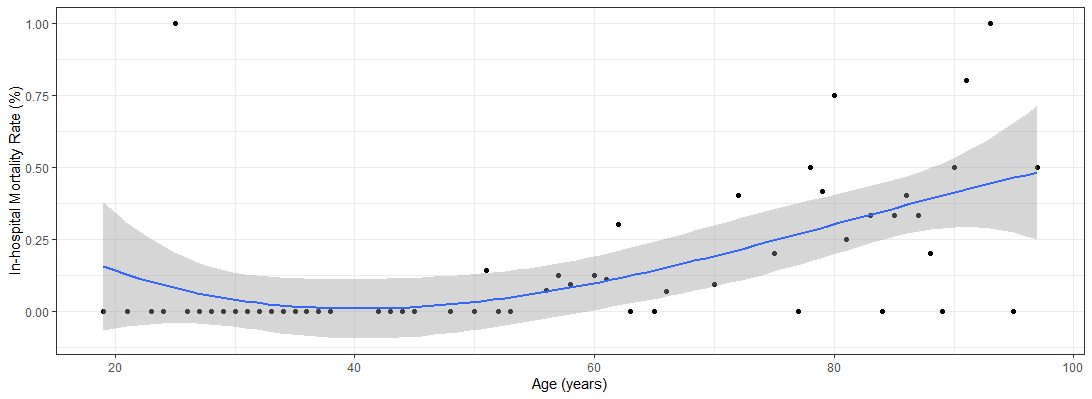

Supplement: Supplementary file 1 [file S0899823X2001435Xsup001.docx]
